# *De Novo* Assembly of the Sea Cucumber *Apostichopus* *japonicus* Hemocytes Transcriptome to Identify miRNA Targets Associated with Skin Ulceration Syndrome

**DOI:** 10.1371/journal.pone.0073506

**Published:** 2013-09-12

**Authors:** Pengjuan Zhang, Chenghua Li, Lin Zhu, Xiurong Su, Ye Li, Chunhua Jin, Taiwu Li

**Affiliations:** 1 School of Marine Sciences, Ningbo University, Ningbo, Zhejiang Province, P. R. China; 2 Ningbo City College of Vocational Technology, Ningbo, P. R. China; Kyushu Institute of Technology, Japan

## Abstract

**Background:**

*De* *novo* transcriptome sequencing is a robust method of predicting miRNA target genes, especially samples without reference genomes. Differentially expressed miRNAs have been previously identified in hemocytes collected from healthy skin and from skin affected by skin ulceration syndrome (SUS) in *Apostichopus* *japonicus* . Target identification for these differentially expressed miRNAs is a major challenge for this non-model organism.

**Methodology/Principal Findings:**

To thoroughly understand the function of miRNAs, a normalized cDNA library was sequenced with the Illumina Hiseq2000 technology. A total of 91,098,474 clean reads corresponding to 251,148 unigenes, each with an average length of 494bp, were obtained. Blastx analysis against a nonredundant (nr) NCBI protein database revealed that in this set, 52,680 unigenes coded for 3,893 annotated proteins. Two digital gene expression (DGE) libraries from healthy and SUS samples showed that 4,858 of the unigenes were expressed at significantly different levels; 2,163 were significantly up-regulated, while 2,695 were significantly down-regulated. The computational prediction of miRNA targets from these differentially expressed genes identified 732 unigenes as the targets of 57 conserved and 8 putative novel miRNA families, including spu-miRNA-31 and spu-miRNA-2008.

**Conclusion:**

This study demonstrates the feasibility of identifying miRNA targets by transcriptome analysis. The DGE assembly data represent a substantial increase in the genomic resources available for this species and will provide insights into the gene expression profile analysis and the miRNAs function annotations of further studies.

## Introduction

RNA-seq is a powerful and rapidly developing approach for unbiased transcriptome analysis. It relies on deep-sequencing technologies and has revolutionized the way in which transcriptomes are studied. Recent advances in RNA-seq allow us to generate an unprecedented global view of the transcriptome and provide a more efficient method to explore the whole transcriptional landscape [1,2]. In addition, the dynamic range, sensitivity and specificity of RNA-seq also make it ideal for quantitatively analyzing various aspects of gene regulation [3]. Therefore, RNA-seq technologies have proven to be efficient and reliable for genome and transcriptome sequencing, and they are suitable for the study of non-model organisms, including economically important marine animals. Compared to other sequencing platforms, such as GS FLX and SOLID, the Illumina sequencing platform produces large amounts of short-read data at a lower cost [4].

*Apostichopus* *japonicus* is one of the most important aquaculture animals. Skin ulceration syndrome (SUS) is the main limitation in the development of *A* *. japonicus* culture industries [5]. Many efforts have been made to uncover the reason for SUS outbreaks in cultured *A* *. japonicus* , and some pathogens responsible for this disease, such as *Vibrio*, *Pseudomonas* and spherical virus, have already been isolated [6-8]. Regarding the immune defense of the sea cucumber, various effectors, such as lectin [9], lysozyme [10,11] and complement component 3 [12], have been isolated and characterized at the mRNA or protein level. However, the connection between pathogen infection and immune-related gene expression is largely unknown.

MicroRNAs (miRNAs) are key effectors in mediating host-pathogen interactions and constitute a family of small RNA species; they are considered to be a promising candidate for regulating the interaction between host and pathogen [13]. Therefore, dissecting the biological functions of miRNAs may help us understand the pathogenic mechanism SUS in *A* *. japonicus* . In our previous study, several differentially expressed miRNAs, such as spu-miRNA-31 and spu-miRNA-2008, have been identified and linked to *A* *. japonicus* SUS outbreaks under natural conditions [14]. To thoroughly interpret the biological functions of these miRNAs, the first step is predicting their targets. However, there is a large gap in target prediction and functional validation between invertebrates’ miRNA and model organisms’ miRNA. Therefore, establishing a more powerful experimental scheme for target identification is preferred in non-model organisms.

Although two parallel *A* *. japonicus* transcriptomes have already been conducted at different developmental stages [15] and in intestine and body wall tissues [16], the data presented here represent the first effort to analyze the transcriptome of the *A* *. japonicus* affected by SUS under natural conditions. First, a normalized cDNA library from the same sample used for our miRNA analysis [14] was constructed and sequenced with Illumina Hiseq2000. Second, the sequence reads were assembled and annotated by a BLAST analysis against the NCBI NR database; third, two digital gene expression (DGE) libraries were sequenced to screen differentially expressed genes and to predict miRNA targets. Our work will provide a fast approach to identify the target genes of some vital miRNAs and to characterize their functional/regulatory network to increase our understanding of SUS outbreaks in this species.

## Materials and Methods

### Ethics Statement

The sea cucumbers ( *A* *. japonicus* ) here are commercially cultured animals, and all the experiments were conducted in accordance with the recommendations in the Guide for the Care and Use of Laboratory Animals of the National Institutes of Health. The study protocol was approved by the Experimental Animal Ethics Committee of Ningbo University, China.

### Sample collection

Thirty juvenile sea cucumbers (3-5 g) suffering from skin ulceration syndrome were sampled from indoor ponds of the Pulandian Hatchery in Dalian, China, in October of 2011. Identical numbers of healthy samples were also collected as a control group. White skin ulceration was the criterion for differentiating diseased individuals from healthy ones. The hemocytes were collected by centrifuging at 1000 ×g, 4 °C for 10 min.

### Normalized cDNA library construction and sequencing

Experimental protocols for the cDNA normalization sequence were performed according to the manufacturer’s technical instructions. Briefly, the total RNA was isolated from hemocytes with TRIZOL reagent (Invitrogen, Grand Island, NY), and the RNA was purified by the Qiagen RNeasy mini kit (Qiagen, Valencia, CA). The purified RNA was analyzed on a ND-8000 spectrophotometer (Nanodrop, Technologies, Wilmington, DE) followed by agarose electrophoresis to determine the quantity. Poly (A) mRNA was purified from the total RNA using oligo (dT) magnetic beads. Equal amounts of the high-quality mRNA samples from each group were then pooled for cDNA library preparation using the TruSeq RNA sample preparation kit (Illumina). The mRNA mix was first fragmented into small pieces with an additional fragmentation buffer, after which the first-strand and double-strand cDNA was synthesized using random hexamer-primers and SuperScript II (Invitrogen, CA, USA). The double stranded cDNA was subsequently purified for end repair, dA tailing, adaptor ligation and sequential DNA fragment enrichment. The concentration of the cDNA library was determined on an Agilent Technologies 2100 Bioanalyzer by Agilent DNA-1000 and diluted to 10 nM. A 2µL aliquot was used to generate clusters on the Illumina Cluster Station using the Paired-End Cluster Generation Kit v2 (Illumina) and was sequenced on the GAII using the SBS 36-cycle Sequencing Kit v3, following the manufacturer’s instructions. Two lanes per group were sequenced as 100-bp reads, and image analysis and base calling were performed with CASAVA1.6.0 (Illumina) according to the manufacturer’s instructions.

### Data processing and assembly

**Figure 1:**
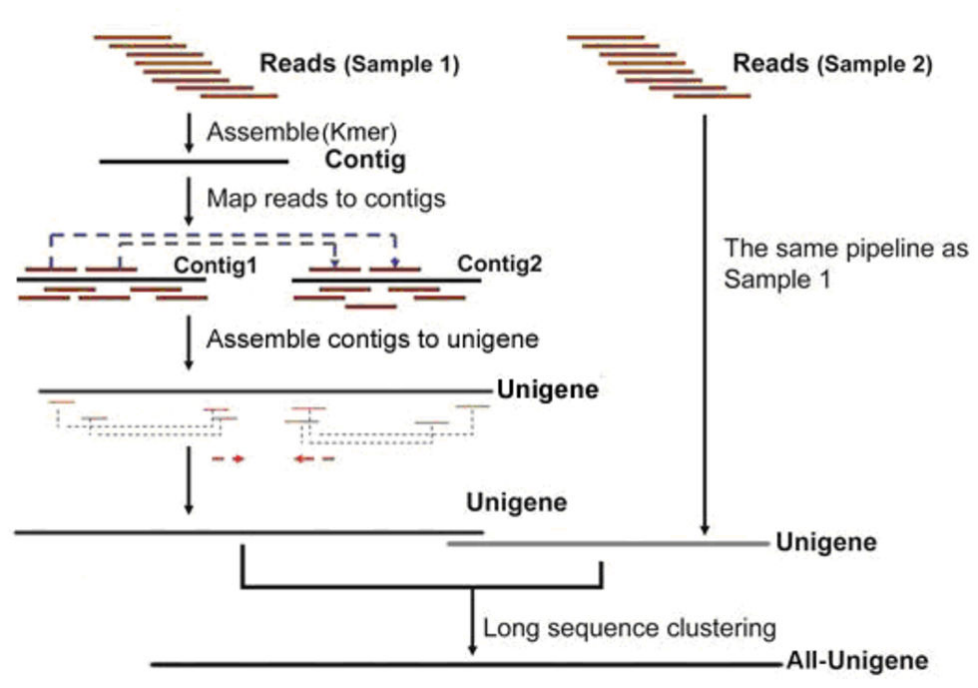
Schematic of the transcriptome assembly analysis.

After the initial image was taken, the data were transformed into sequence data, the raw reads were then cleaned by removing the adaptor sequences and any ambiguous or low quality reads. *De novo* transcriptome assembly was carried out with the short-read assembly program Trinity, as follows [17]: first, short reads were assembled into high-coverage contigs that could not be extended farther in either direction in a k-mer-based approach for fast and efficient transcript assembly. Then, the related contigs were clustered and a de Bruijn graph for each cluster was constructed. Finally, in the context of the corresponding de Bruijn graph and all plausible transcripts, alternatively spliced isoforms and transcripts were derived. The detail process is shown in Figure 1.

Blastx analysis of unigenes longer than 200bp was conducted against the Uniprot database (www.uniprot.org), the Non-Redundant database (www.ncbi.nlm.nih.gov/protein), the COG database (www.ncbi.nlm.nih.gov/COG/) and the KEGG database (http://www.genome.jp/kegg/), with an E value of 0.001. The Best Blast Hit from all Blast results was parsed for a homology-based functional annotation. GO annotation was performed based on the annotation information from Uniprot, using Blast2GO (http://www.balst2go.org/). The classification of GO functions was conducted using R software.

### Sequencing the DGE (digital gene expression) library

RNA was extracted separately from the healthy and SUS samples in the cDNA library construction. Each mRNA, taken from approximately 10µg of total RNA, was treated as described above. The required fragments were purified by agarose gel electrophoresis and enriched by PCR amplification. The library products were then sequenced via Illumina HiSeq 2000 using paired-end technology in a single run.

### Differentially expressed genes between the two DGE libraries

Low-quality reads were omitted from data analysis. High-quality reads were mapped to reference sequences (unigenes from the transcriptome data of the normalized cDNA library) using Top hat [18]. Gene expression levels were calculated using the Fragments Per Kilobase of transcript per Million fragments mapped (FPKM) method. The calculation of unigene expression levels and the identification of unigenes that were differentially expressed between the libraries were performed by Cufflinks [19] based on different normalization patterns. The normalization pattern of the unigene expression level calculation was a total-hits-norm as well as a normalization pattern of differentially expressed unigenes, while the calculation followed a compatible-hits-norm [20]. For the significance analysis of the differentially expressed unigenes, the false discovery rate (FDR) method was used to determine the q-value threshold in multiple tests [21]. The significance of differences in gene expression was screened using a threshold q<0.05. Then, the differentially expressed genes across the samples were further annotated by GO and KEGG pathway analysis with a hypergeometric test.

### miRNA target prediction

Computational identification of differentially expressed miRNA targets was performed using the miRanda toolbox [22] using the complementary region between miRNAs and mRNAs and the thermodynamic stability of the miRNA-mRNA duplex. All the mRNAs used for target prediction came from the differentially expressed unigenes obtained above. The miRanda toolbox utilized a dynamic programming algorithm to search the complementary regions between the miRNA and the 3'UTR of the mRNA, and the scores were based on sequence complementary as well as minimum free energy of RNA duplex and were calculated with the Vienna RNA package [23]. All detected targets with scores and energies less than the threshold parameters of S>90 (single-residue pair scores) and ΔG<-17 kcal/mol (minimum free energy) were selected as potential targets.

## Results and Discussion

Since 2003, skin ulceration syndrome (SUS) has been frequently observed in *A* *. japonicus* farms. This disease produces ulcers in integument muscles, peristome tumescence, autolysis and death and has severely limited further development of this important industry in China [6]. Antagonistic bacteria, such as *Pseudoalteromonas* *elyakovii* and *Vibrio ordalii*, enhance non-specifically immune-related enzyme activities and disease resistance in sea cucumbers and provide a theoretical basis for *A* *. japonicus* disease prevention and healthy aquaculture [24]. Nonetheless, the molecular mechanism of this disease outbreak is still far from fully understood. The identification and characterization of candidate genes or miRNAs involved in SUS outbreaks would represent the first step in understanding the genetic basis of this process in sea cucumbers.

### *De novo* assemblies and unigenes annotation

**Figure 2:**
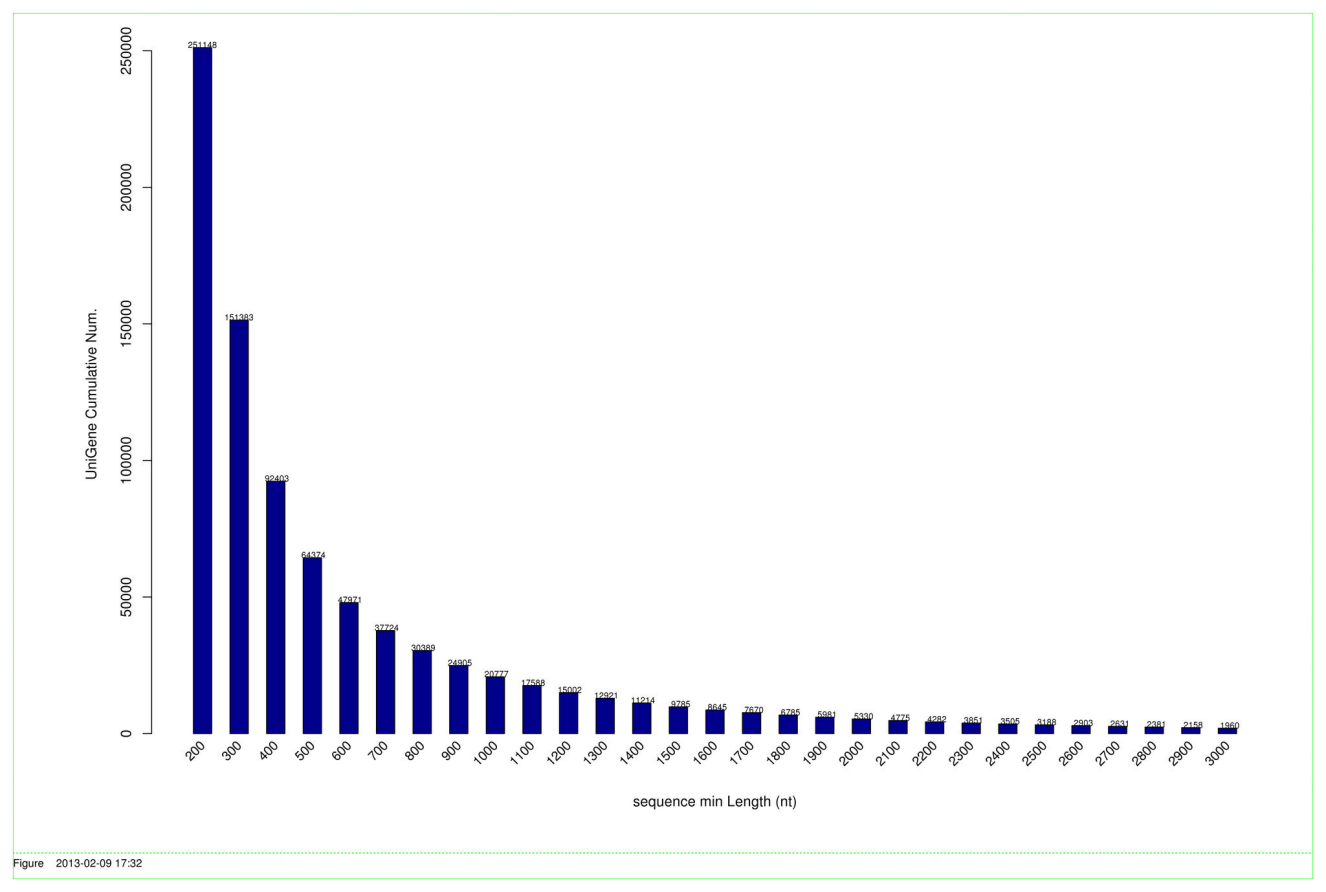
The length distribution of assembled unigenes in the sequenced normalized cDNA library.

A normalized cDNA library from diseased and control sea cucumber hemocytes was constructed and paired-end sequenced by Illumina in a single run. A total of 93,476,668 sequencing reads were generated. After trimming the adapter sequences and removing low-quality sequences, 91,098,474 clean reads remained for the normalized cDNA library, with average GC content of 38.91%. The raw sequencing reads have been submitted to NCBI Short Read Archive under the accession number of SRA080354. These short reads were further assembled into 251,148 unigenes with an average length of 494 bp (Table 1). The size distribution of these unigenes ranged from 201 to 19,766 bp, and 5,330 were larger than 2,000 bp (Figure 2).

**Table 1:** Summaries of sequencing cDNA library.

Total reads	93,476,668
Clean reads	91,098,474
Reads length (bp)	101
Average GC content (%)	38.91%
Number of unigenes	251,148
Total length of unigenes (bp)	124,142,187
Mean length of unigenes (bp)	494
N50 of unigenes (bp)	549
Maximal length of unigenes (bp)	19,766
Sequences with E-value,10^-3^	52,680

### Annotation of predicted proteins

**Figure 3:**
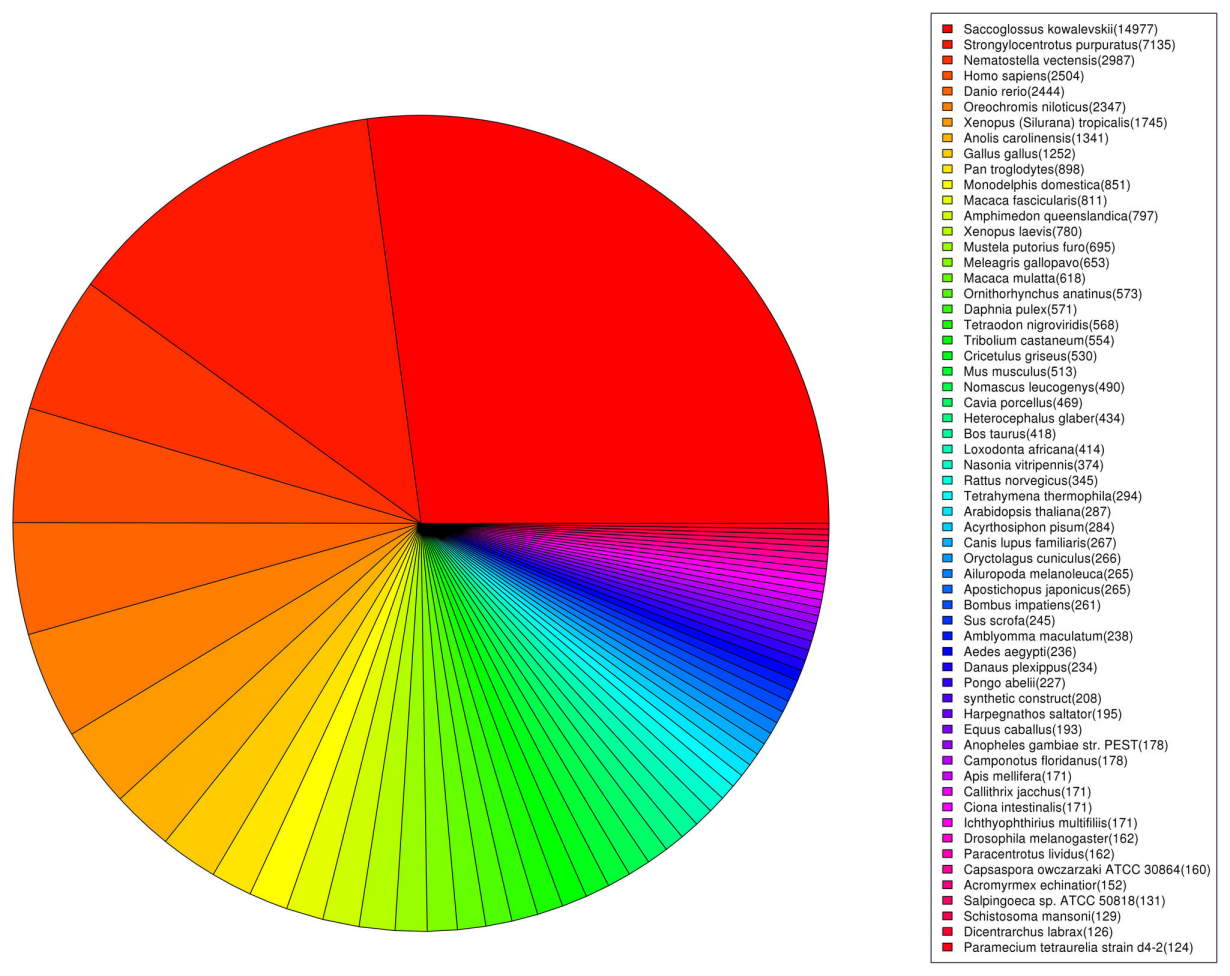
Species distribution of the Blastx matches of the transcriptome unigenes.

Unigene sequences were annotated by searching the Uniprot database combined with the nonredundant (nr) NCBI protein database using BLASTx with a cutoff E-value of 10^-3^. A total of 52,680 distinct sequences (20.98% of the unigenes) matched known genes corresponding to 3,893 of the annotated proteins (Table S1). The order of majority of sequence homology was *Saccoglossuskowalevskii* (25.11%) and *Strongylocentrotus purpuratus* (11.96%), a model organism in Echinodermata (Figure 3).

### Functional annotation of unigenes

**Figure 4:**
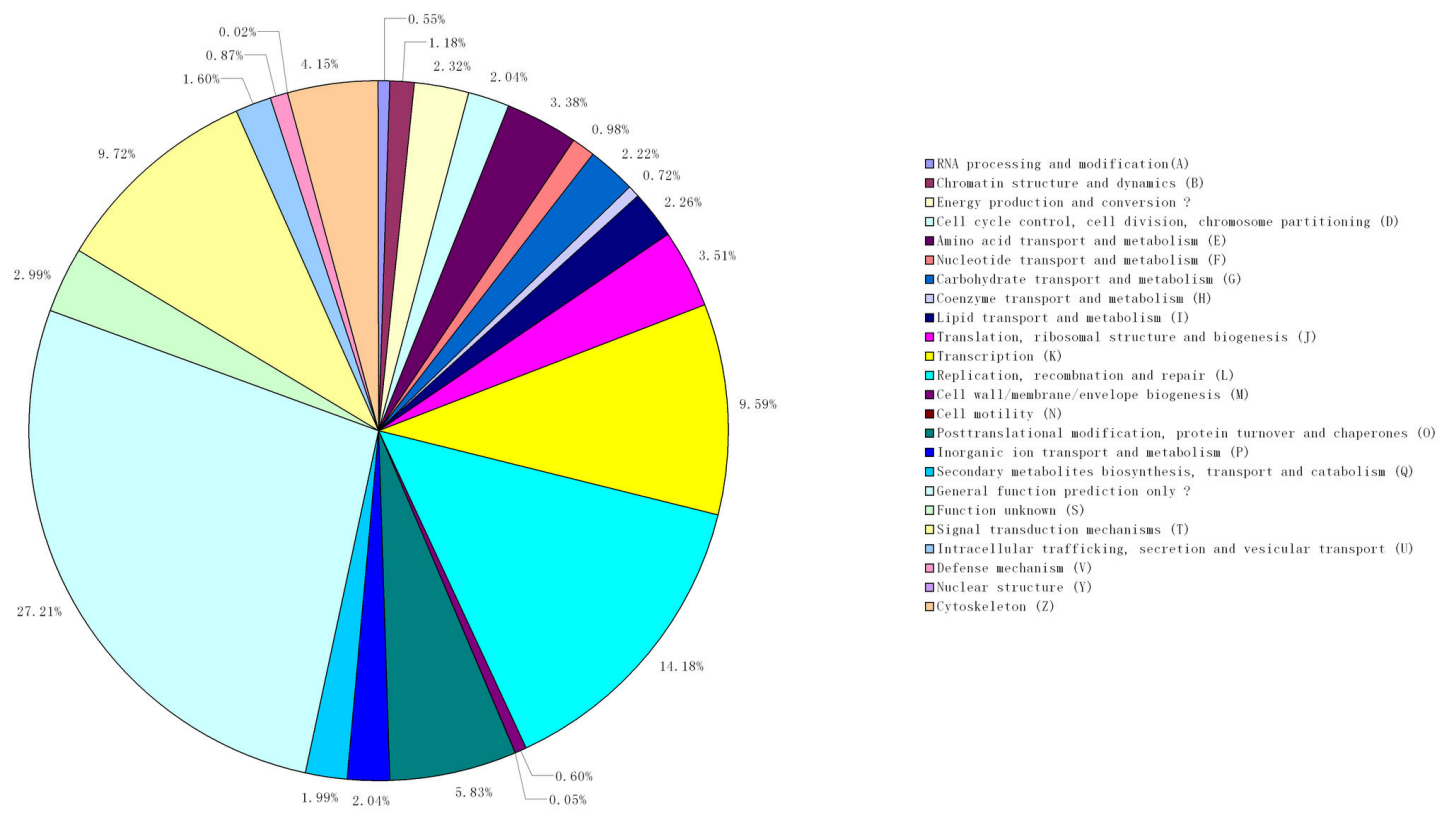
Classification of clusters of the orthologous groups (COG) of the transcriptome unigenes.

Assignments of clusters of orthologous groups (COG) were used to predict and classify the possible functions of these unigenes. Based on sequence homology, 36,407 unigenes (69.11%) were annotated and divided into 25 specific categories (Figure 4). The general function category, which contained 14,180 unigenes (27.21%), was the largest, followed by the replication, recombination and repair (14.18%), signal transduction mechanisms (9.72%) and transcription (9.59%) categories. Notably, no protein involved in the extracellular structure (W) was detected in our study.

### GO annotation and KEGG pathway analyses

**Figure 5:**
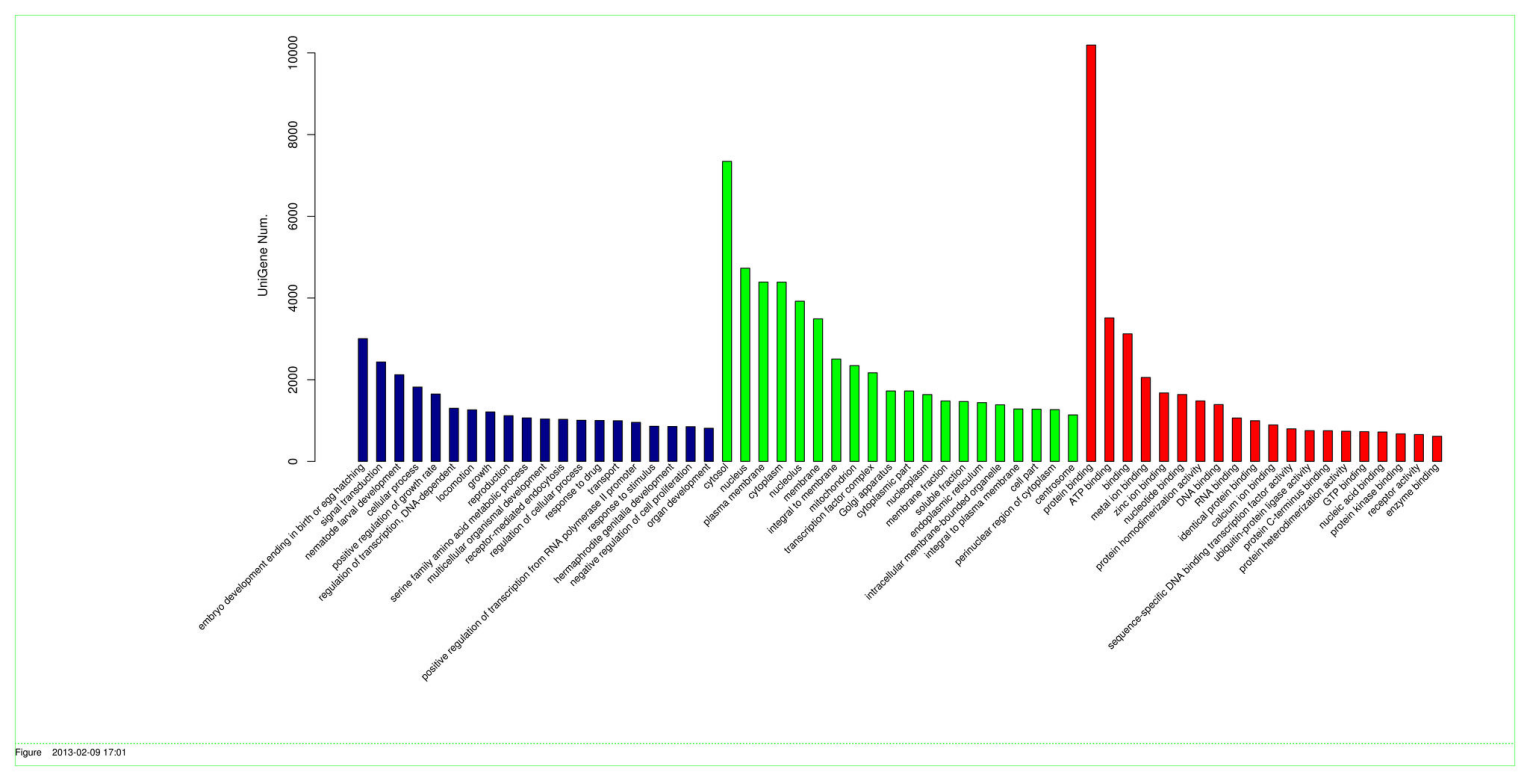
Classification of the gene ontology (GO) for the sea cucumber transcriptome.

Using the Uniprot protein database as a reference, *A* *. japonicus* transcripts could be assigned to three categories: biological processes, molecular functions and cellular components. Among the various biological processes, embryo development ending in birth or egg hatching (3,006) and signal transduction (2,434) as well as nematode larval development (2,122) were the most highly represented members (Figure 5). Important functions, such as drug (1003) and stimulus (862) responses, were also identified in this category. Similarly, cytosol (7,345) and protein binding (10,192) were the most represented in the cellular component and molecular function categories, respectively.

Searching against the Kyoto Encyclopedia of Genes and Genomes Pathway database (KEGG) indicated that 11,134 unigenes mapped to 320 KEGG pathways. The well-represented pathways were the metabolic pathway (827) and the biosynthesis of secondary metabolites pathway (207). Meanwhile, some pathways related to immune response were also identified, such as the NF-kappaB signaling pathway (45) and the cytokine-cytokine receptor interaction pathway (53) (Table S2).

### Digital gene expression (DGE) library sequencing

Based on the transcriptome sequence data, two DGE libraries were constructed to identify the differentially expressed unigenes between the normal and SUS samples. After removing low-quality reads, 35,157,708 and 31,882,637 clean reads were generated from the control and SUS libraries, respectively (Table 2). Among these clean reads, 12,157,026 of the control and 11,735,161 of the SUS reads were mapped to unigenes in the normalized library (Table 2).

**Table 2:** Alignment statistics of the DGE analysis.

	Control library	SUS library
Total reads	35,157,708	31,882,637
Total mapped reads	20,456,845	19,037,315
Unique match reads	12,157,026	11,735,161
Multiple match reads	8,299,819	7,302,154
Toal unmapped reads	14,700,863	12,845,322

### Differential gene expression between the SUS and control libraries

**Figure 6:**
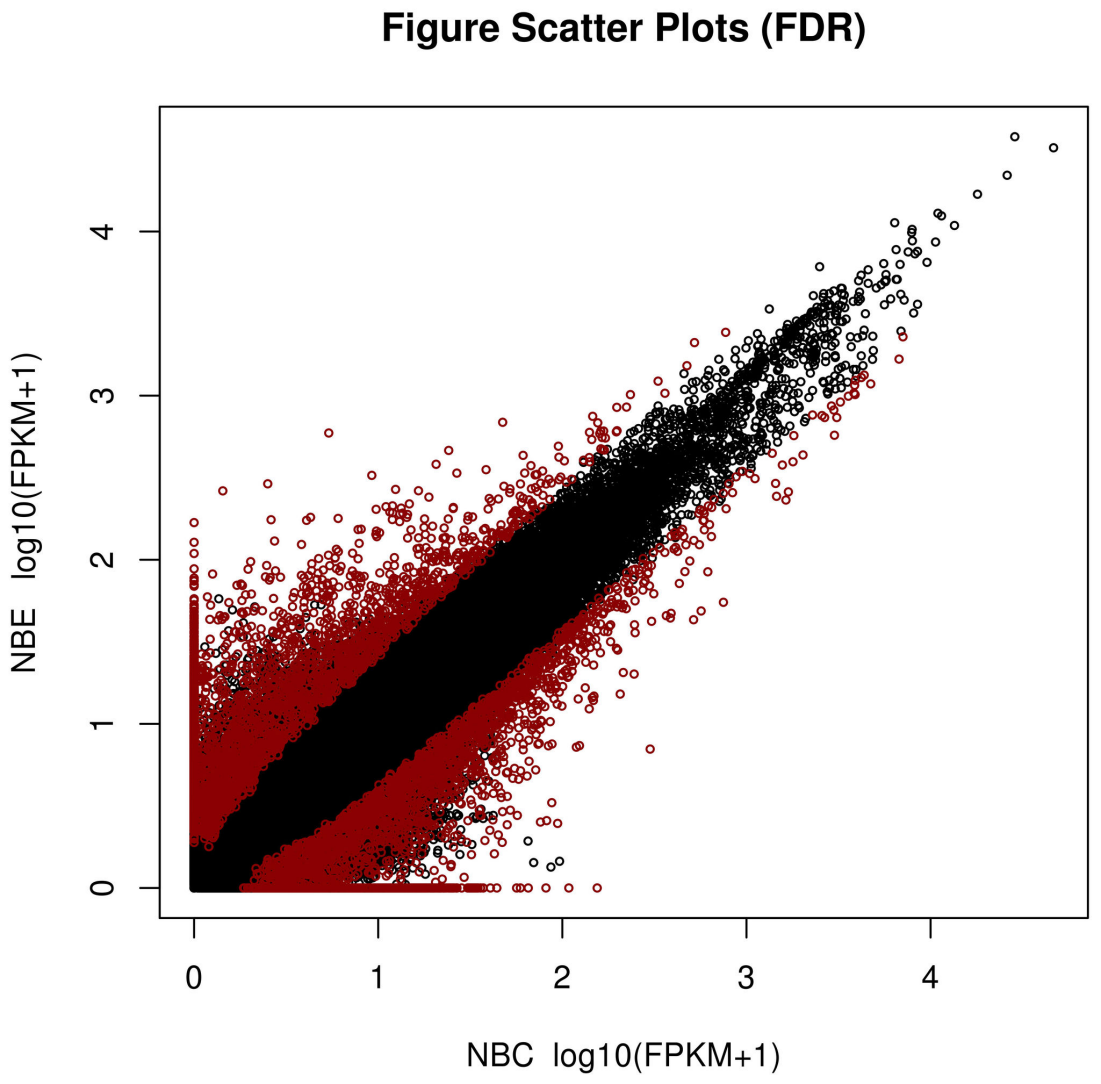
Differentially expressed transcripts between the control and SUS sea cucumber samples using the false discovery rate (FDR) method.

**Figure 7:**
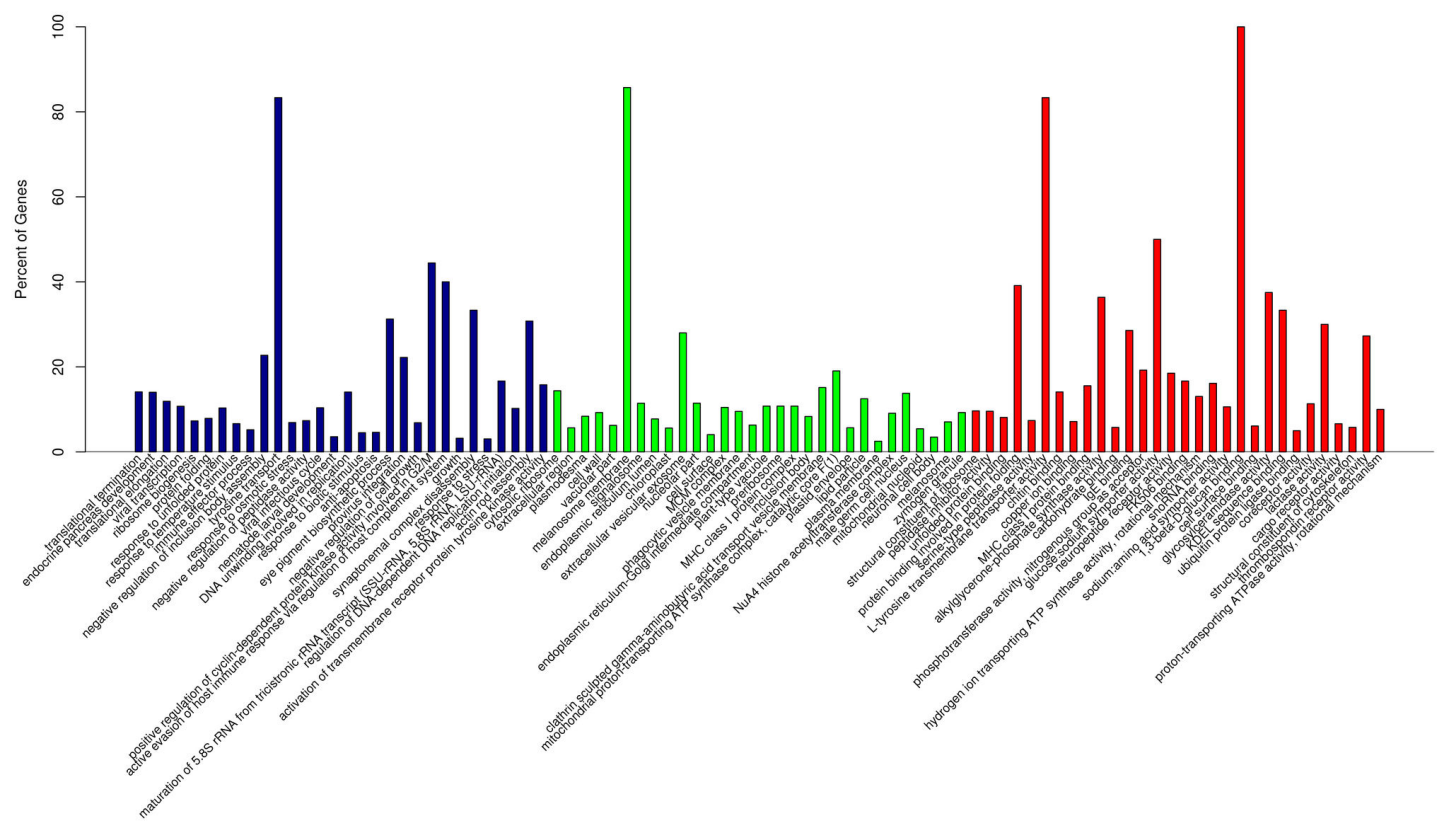
The top thirty differentially expressed transcripts enriched by GO terms.

Genes found to have significant differences in their expression were identified according to the FPKM model and are shown in Figure 6. The results suggest that the expression of 4,858 genes was significantly different between the diseased and control sea cucumbers. Of these genes, 2,163 were up-regulated and 2,695 were down-regulated in the control compared to the SUS samples (Table S3). GO enrichment analysis of DEGs indicated that these genes were significantly enriched in tyrosine transport (biological process), melanosome membrane (cellular component), and L-tyrosine transmembrane transporter activity and 1-3-beta-glycan binding (molecular function) (Table S4). The top thirty enriched GO terms are presented in Figure 7. Pathway enrichment analysis found DEGs mainly enriched in nitrogen metabolism, chloroalkane and choroalkene degradation (Table S5). Notably, several genes involved in the immune and inflammatory response were also identified and ascribed by type, such as alpha-2 macroglobulin [25,26], the mannose receptor [27], and cathepsin B [28].

### miRNA target prediction

The identification of miRNAs and their targets is important for understanding the physiological functions of miRNAs and the functional roles of differentially expressed miRNAs between healthy and diseased *A* *. japonicus* . We therefore were most interested in predicting miRNA target genes involved in the immune response or immune system, according to KAAS analysis. In our previous study, small RNAs deep-sequencing data were aligned with miRBase18.0 to search for known miRNAs with complete matches, namely, conserved miRNAs. Reads that did not match the miRBase database were marked as unannotated and analyzed by alignment with the sea urchin genomic sequence (http://www.spbase.org/SpBase/download/). Meanwhile, miRNAs predicted by miRDeep2.0, which could form stable secondary structures, were identified as novel miRNAs [14]. The computational prediction of miRNA targets from differentially expressed genes led to the identification of 732 unigenes as the predicted target genes of 57 conserved and 8 putative novel miRNA families, with an average of approximately 11 targets per miRNA. To further understanding the miRNA-gene regulatory network, the identified target genes involved in biological processes, molecular functions, and cellular components were defined using GO annotations. GO analysis demonstrated that these targets were involved in a broad range of physiological processes, including gene expression, transcription regulation, the immune system process, and the response to stress or stimulus (Table S6).

As a key players in the SUS outbreak, the targets of spu-miR-31 and spu-miR-2008 were further analyzed in the present study. We found that a number of the predicted targeted genes of spu-miR-31 were associated with signaling pathways in the immune response process (Table S5), including alpha-2-macroglobulin (A2M), the mannose receptor (MR) and heat shock protein 90 kDa-beta. All identified genes showed clearly down-regulated expression profiles in the SUS group compared to the control group (Table S3). The decreased expression profile of these targets in the diseased samples supported our previous finding that the expression of spu-miRNA-31 was significantly up-regulated, and the read count was 891 and 1705 in the control and SUS groups, respectively (data unpublished).

A2M is a non-specific protease inhibitor involved in the host defense of both invertebrates and vertebrates. It has been reported that A2M plays an important role in the innate immune response to *Vibrio* pathogen infection [25,26]. Most importantly, this protein serves as a complement-like opsonin and promotes phagocytosis of some Gram-negative bacteria in a mosquito hemocyte-like cell line [29]. All these functional analyses are consistent with the fact that Gram-negative bacteria are the major pathogen responsible for SUS outbreaks in sea cucumbers [6,7]. Mannose receptor (MR), a C-type lectin family member, is essential for both pro-inflammatory and anti-inflammatory cytokine production. In higher animals, LPS-induced macrophage activation is accompanied by the down-regulation of mannose receptors [30], which has been confirmed by Shepherd et al [31].

Notably, heat shock protein (HSP) 90 kDa-beta was predicted as a target for not only spu-miR-31 but also for spu-miR-2008, suggesting that multiple miRNAs can regulate a single gene. The co-regulation of HSP90 by the two miRNAs was speculated to be related to its broad-spectrum activity, and HSPs were demonstrated to be expressed as stress proteins in response to a wide range of abiotic and biological stressors, including infectious pathogens. Concerning their immunological roles, HSPs are potent activators of the innate immune system [32,33]. It has been reported that T cell responses to HSPS have disease suppressive activities through the production of anti-inflammatory cytokines in patients and in models of inflammatory diseases [34]. To inhibit innate immunity and ensure successful infection, pathogens have evolved to employ host miRNAs to control the expression of HSPs at a lower level.

In summary, the discovery of miRNAs has introduced a new paradigm into the current understanding of gene regulatory systems. The identification of miRNA target genes is believed to be an important step in understanding the role of miRNAs in gene regulatory networks. As part of the effort to understand interactions between miRNAs and their targets, computational algorithms have been developed according to observed rules for features such as the degree of hybridization between the two RNA molecules [35,36]. However, while such in silico approaches for miRNA target identification can be certainly useful, they still have many problems that need to be solved. In particular, the problem of target prediction is compounded when considering context-specific targeting. Whether a gene and its associated miRNA are expressed in the same sample will certainly influence the accuracy of the predicted target, but several recent papers also suggest that the expression of other genes targeted by the same miRNA influence how well that miRNA binds to its other targets. Single nucleotide polymorphisms in miRNA target sites will also affect the interaction between the miRNAs and the mRNAs [37,38]. In combination, the above issues make it very difficult to accurately predict miRNA targets. Fortunately, several studies have suggested that the simultaneous expression profiling [39] or inverse expression relationship between miRNAs and mRNAs [40] are effective strategies for more reliably identifying miRNA-target mRNA pairs in large sets of transcriptome experiments [41,42]. To decrease the false positive rates of this study, the RNA-seq and miRNA-seq were conducted in the same biological sample. Likewise, to downsize the number of putative target genes, the miRNA targets were predicted from differentially expressed genes. Even so, there still exist inevitable false positive predictions. Fortunately, many experimental approaches to validate miRNA targets have become more refined and more economically feasible to conduct [43,44]. Further study will focus on the experimental validation of interesting miRNAs.

## Supporting Information

Table S1 **Summary of annotated unigenes against nonredundant (nr) NCBI protein database.**
(XLS)

Table S2 **KEGG annotation of all unigenes.**
(XLS)

Table S3 **Summary differential expressed genes.**
(XLS)

Table S4 **GO Enrichment of differentially expressed genes.**
(CSV)

Table S5 **Pathway Enrichment of differentially expressed genes.**
(CSV)

Table S6 **Summary of predicted miRNAs targets.**
(XLSX)
